# Ligand-Free Nano-Au Catalysts on Nitrogen-Doped Graphene Filter for Continuous Flow Catalysis

**DOI:** 10.3390/nano8090688

**Published:** 2018-09-05

**Authors:** Yanbiao Liu, Xiang Liu, Shengnan Yang, Fang Li, Chensi Shen, Chunyan Ma, Manhong Huang, Wolfgang Sand

**Affiliations:** 1 Textile Pollution Controlling Engineering Center of Ministry of Environmental Protection, College of Environmental Science and Engineering, Donghua University, Shanghai 201620, China; ninjiaxiang@gmail.com (X.L.); ysnshengnan@gmail.com (S.Y.); lifang@dhu.edu.cn (F.L.); shencs@dhu.edu.cn (C.S.); machunyan@dhu.edu.cn (C.M.); huangmanhong@dhu.edu.cn (M.H.); wolfgang.sand@uni-due.de (W.S.); 2 Shanghai Institute of Pollution Control and Ecological Security, Shanghai 200092, China; 3 Institute of Biosciences, Freiberg University of Mining and Technology, 09599 Freiberg, Germany

**Keywords:** catalytic filter, nitrogen-doped graphene, surfactant-free, Au nanoparticles, continuous flow catalysis

## Abstract

In this study, the authors rationally designed a high-performance catalytic filter for continuous flow catalysis. The catalytic filter consisted of ligand-free nanoscale gold (nano-Au) catalysts and nitrogen-doped graphene (N-rGO). The Au catalyst was fabricated in situ onto a pre-formed N-rGO support by the NaBH_4_ reduction of the Au precursor, and the size of the nano-Au was fine-tuned. A hydrothermal pretreatment of graphene oxide enriched nitrogen-containing species on the surface of two-dimensional graphene supports and enhanced the affinity of Au precursors onto the support via electrocatalytic attraction. The nano-Au catalysts acted as high-performance catalysts, and the N-rGO acted as ideal filter materials to anchor the catalysts. The catalytic activity of the as-designed catalytic filter was evaluated using 4-nitrophenol (4-NP) hydrogenation as a model catalytic reaction. The catalytic filters demonstrated superior catalytic activity and excellent stability, where a complete 4-nitrophenol conversion was readily achieved via a single pass through the catalytic filter. The as-fabricated catalytic filter outperformed the conventional batch reactors due to evidently improved mass transport. Some key operational parameters impacting the catalytic performance were identified and optimized. A similar catalytic performance was also observed for three 4-nitrophenol spiked real water samples (e.g., surface water, tap water, and industrial dyeing wastewater). The excellent catalytic activity of the nano-Au catalysts combined with the two-dimensional and mechanically stable graphene allowed for the rational design of various continuous flow catalytic membranes for potential industrial applications.

## 1. Introduction

Noble metal nanoparticles have been extensively explored for application in catalysis [1,2,3,4,5]. Being small, they offer unique physicochemical properties compared with their bulk counterparts [6,7,8]. The small size means a large surface energy. Unfortunately, these nanoparticles tend to agglomerate to reduce their surface-to-volume ratio and their catalytic activity, and thus are undesirable for industrial applications [9,10].

Nanoparticles can be used directly as a colloid form for catalytic reactions [11,12]. In this case, surface-capping ligands are indispensable for the colloidal stability. However, these ligands often limit surface accessibility of the catalyst by blocking surface active sites, and decrease selectivity and activity by interacting with intermediates [13,14,15,16,17]. The presence of undesirable co-existing ions and natural organic matters may cause their agglomeration as well. Furthermore, the recycling of these nanoscale catalysts from the reaction medium is of importance [18]. Current established post-separation methods such as centrifugation and filtration are unsuitable for these nanoparticles and often lead to serious agglomeration [19].

To address the recycling issue, nanocatalysts can be anchored onto low-cost support materials. In this respect, carbon material [13,20,21,22], metallic oxide [23,24], and polymer, among others [25,26,27], have been employed as effective support materials to accommodate these metallic nanocatalysts. It is noteworthy that the resulting catalytic properties of these nanocomposite materials depend on both the characteristics of nanoparticles and the properties of support materials. For example, the nanoparticles must securely bonded with the support, and the support materials and/or protecting ligands should not block the active sites on the nanoparticles. Dispersing the supported composite catalysts in a reaction medium ensures their accessibility to substrates and allows for post-separation using a conventional filtration, centrifugation or magnetic process. However, the loss and agglomeration of the catalysts can still occur during this tedious process. An ideal way to fully utilize these nanocomposite materials is to load the nanocatalysts onto an immobilized support (e.g., column reactor and fix bed reactor) via continuous flow catalysis [28]. Such a design is attractive for industrial applications due to its high operability and easy controllability. Unfortunately, mass transport-limited kinetics within either column reactors or fix bed reactors packed with immobilized catalysts is still not desirable, which greatly restricts the wide application of these processes. This limitation may be due to the pores that are blocked by the catalyst reactants and/or products. Alternatively, a more promising design is to construct a catalytic membrane reactor by integrating the catalytic nanoparticles with a desirable membrane material [29]. These nanoscale catalysts can be effectively immobilized onto or into a support matrix without inhibiting access to catalytic sites [30]. Such a catalytic membrane reactor ensures that separation and catalytic reactions occur in a single unit and can overcome the sluggish kinetics and improve the mass transport performance [31]. Meanwhile, transforming the unique characteristics of individual nanoscale components into macroscopic materials such as membranes or sheets remains a challenge, as the engineering of these structures often compromises their intrinsic properties [32].

For this contribution to research, ligand-free nanoscale gold (nano-Au) catalysts with a tunable size were grown in situ onto a nitrogen-doped graphene (N-rGO) support membrane for continuous flow catalysis applications. To the best of the authors’ knowledge, only a very limited number of reports are available on the integration of two-dimensional materials with ligand-free nano-Au for continuous flow catalytic applications [33]. Both ligand-free nano-Au and N-rGO supports played an indispensable role in this design. In particular, the nano-Au served as a high-performance catalyst and provided sufficient active sites for catalytic reactions. The graphene or rGO served as a promising candidate for next-generation membrane material. After doping with nitrogen, the as-produced N-rGO could effectively anchor the Au precursors carrying an opposite charge via electrostatic attraction. The catalytic performance was evaluated using the reduction of 4-nitrophenol (4-NP) to 4-aminophenol (4-AP) as a model reaction due to its affirmatory characterization protocol and simplicity. The catalytic activities of the as-fabricated filters were comparatively studied with a conventional batch reactor and the impact of several operational parameters were identified. Finally, a few 4-NP spiked real water samples were challenged with the catalytic filter.

## 2. Experimental Section

### 2.1. Chemicals and Materials

All the reagents were of analytical grade and used without further purification. Sodium borohydride (NaBH_4_, ≥98%) and gold (III) chloride trihydrate (HAuCl_4_·3H_2_O, ≥49% Au basis) were purchased from Sigma-Aldrich (Shanghai, China). 4-Nitrophenol (4-NP, ≥99%) was purchased from Alfar Aesar (Haverhill, MA, USA). The ammonia solution (NH_3_·H_2_O, 25–28%), *N*-methyl-2-pyrrolidone (NMP, ≥99.5%), and ethyl alcohol were purchased from Sinopharm Chemical Reagent Co., Ltd. (Shanghai, China). Graphene oxide powder was provided by Graphene Nanotech Co., Ltd. (Suzhou, China). Deionized water (DI-H_2_O) with a resistivity of 18.2 MΩ·cm was produced from a Milli-Q Direct 8 purification system (Merck, Darmstadt, Germany).

### 2.2. Catalytic Membrane Reactor Fabrication

The N-rGO filters were fabricated according to a reported protocol [34]. Briefly, N-rGO hydrogel was firstly prepared by hydrothermal treatment of a 2.0 mg/mL GO and 2.0 mL NH_3_·H_2_O solution at 180 °C for 20 h. The N-rGO filter was then fabricated by dispersing 0.25 mg/mL of the as-prepared N-rGO hydrogel in NMP by ultrasonication, followed by vacuum filtration of the dispersion solution onto a 5 μm Millipore JMWP PTFE filter (Merck, Darmstadt, Germany). Then, the as-fabricated N-rGO filter was washed sequentially with 100 mL ethanol and 100 mL DI-H_2_O. As a control, an rGO filter was fabricated in a similar procedure without the addition of NH_3_·H_2_O during the hydrothermal process.

The Au/N-rGO catalytic filters were fabricated by the in situ reduction of gold salt. In a typical synthetic process, a 50 mL of 0.04 mmol/L HAuCl_4_ solution and a 50 mL of 0.50 mmol/L NaBH_4_ solution were sequentially passed through the as-fabricated N-rGO filter using a 4-channel Ismatec REGLO digital peristaltic pump (Wertheim, Germany) at a flow rate of 1.0 mL/min.

### 2.3. Characterizations

The morphologies of the as-fabricated samples were examined on a JSM-6700F field emission scanning electron microscopy (FESEM, JEOL, Peabody, MA, USA) and a 2100 transmission electron microscopy (TEM, JEOL, Peabody, MA, USA). X-ray photoelectron spectroscopy (XPS) spectra were performed on a Escalab 250Xi XPS system (Thermo Fisher Scientific, Waltham, MA, USA), where the analysis chamber was 10^−9^ Torr and the bonding energies were calibrated and referenced to C 1s line at 284.8 eV. The Raman characterization of the filters was recorded on a LabRAM HR Evolution Raman spectrometer (Horiba, Kyoto Japan) excited by a 633 nm radiation from an He-Ne laser. The crystallinity of the filters was characterized by a SmartLab X-ray diffraction (XRD, Rigaku, Tokyo, Japan).

### 2.4. Catalytic Activity Evaluation

The catalytic activity of the catalytic filters was evaluated using the conversion of 4-nitrophenol (4-NP) to 4-aminophenol as a model reaction. The catalytic filters were first loaded into a polycarbonate Whatman filter casing (D = 47 mm, Piscataway, NJ). The effective active area of the catalytic membrane was 7.1 cm^2^. A 4-channel Ismatec REGLO digital peristaltic pump (Cole-Parmer GmbH, Germany) was used to pump freshly-prepared 4-NP and NaBH_4_ solution through the filter. The liquid residency time within the catalytic reactor was only 0.3 s. A photograph of the operational device is shown in Figure S1. Effluents were collected and analyzed by a UV-2600 UV-vis spectrometer (Shimadzu, Kyoto, Japan). As a comparison, this reaction was performed in a conventional batch reactor system using the same catalytic filter in 60 mL of 0.2 mmol/L 4-NP and 37.5 mmol/L freshly-prepared NaBH_4_ solution. The 4-NP conversion was calculated using pseudo-first-order kinetics due to the large excess of NaBH_4_ in the reaction medium.

## 3. Results and Discussion

**Figure 1:**
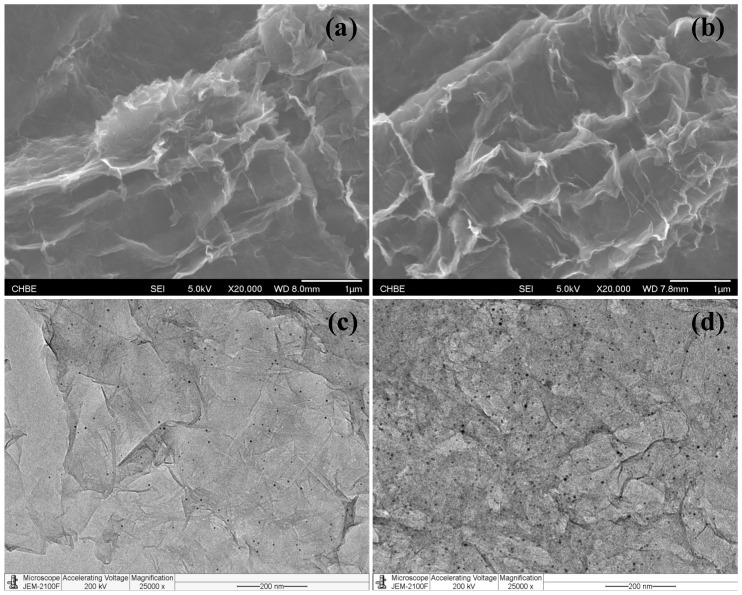
Field emission scanning electron microscopy (FESEM) and transmission electron microscopy (TEM) characterizations of gold/nitrogen-doped graphene (Au/N-rGO) filter at an Au/C ratio of (**a**,**c**) 0.1% and (**b**,**d**) 0.2%, respectively.

Using the typical synthesis process of a catalytic filter, a certain amount of Au precursors was passed through a pre-fabricated N-rGO filter. An in situ reduction of the Au precursors was then carried out by flowing an excess amount of NaBH_4_ solution to yield nano-Au catalysts. Figure 1 displays the N-rGO filter and Au/N-rGO filters prepared with different Au precursor concentrations. As has been previously reported, the N-rGO filter shows a crumpled sheet-like surface, typical of graphene [35]. The space between the sheets provides microchannels for the passage of fluids. As can be seen, the particle size and the loading amount of nano-Au can be tuned by varying the concentration of Au precursors. At a limited Au/C weight ratio of 0.1% and 0.2%, no visible particles were observed from the FESEM characterizations. However, a TEM characterization of these same samples showed that well-defined nano-Au with an average diameter of 2.0 ± 0.5 nm and 6.2 ± 1.1 nm, respectively, were formed and both uniformly distributed onto the N-rGO surface (see Figure 1c,d). Aggregates of Au particles appeared when the Au/C ratio reached ≥0.5% (see Figure S2, Supplementary Materials). For example, the average diameter of Au particles was identified as 75 ± 16 nm and 350 ± 35 nm, respectively, at an Au/C ratio of 2.0% and 5.0%. This result showed that supported ligand-free Au nanoparticles with tunable size could be prepared in situ onto an N-rGO support. The obtained ligand-free AuNPs had more active sites exposed and were expected to be exceptionally active in selected catalytic reactions. Moreover, the wrinkled surface morphologies of the N-rGO remained after loading the Au catalyst. This suggested that the Au loading process through in situ reduction did not greatly affect the N-rGO’s microstructure. Energy-dispersive spectroscopy (EDS) analysis also supports the successful loading of nano-Au onto N-rGO filters, with a typical Au atomic ratio of 0.5–3.3% at an Au/C ratio of 1–5% (see Table S1, Supplementary Materials).

Another interesting finding is that more nano-Au catalysts were produced on an N-rGO surface compared with that of an rGO counterpart. This may be due to the nucleation of nanocatalysts on the support, which is related to some extent to electrostatic interactions. To confirm this hypothesis, a zeta potential measurement of the N-rGO aqueous solution was performed. Indeed, a positive zeta potential of ζ = +0.4 mV was obtained, whereas a negative zeta potential of rGO (ζ = −1.2 mV) was obtained under similar conditions. This can be ascribed to the successful doping of nitrogen into the rGO. It is well known that graphene oxide is negatively charged due to its many oxygen-containing functional groups such as carbonyl, hydroxyl, and carboxyl [36]. A positive zeta potential of the N-rGO was obtained after hydrothermal treatment of the GO dispersion solution, employing overheated supercritical water and NH_3_.H_2_O acting as a reducing agent. However, the as-produced rGO was still negatively charged due to the presence of the remaining oxy-functional groups. This also suggests that the GO reduction using ammonia and supercritical water as reducing agents (N-rGO case) is more thorough than using supercritical water alone (rGO case). Therefore, the electrostatic attraction between the positively charged nitrogenous groups in N-rGO and the negatively charged [AuCl_4_] ions provided an effective driving force for the formation and stabilization of these Au precursors. After doping with N, more nucleation sites were available on the graphene surface. This favors the anchoring of Au precursors via electrostatic attraction during their passing through the N-rGO filter, and the further formation of nano-Au by NaBH_4_ reduction. Previous studies have reported that N-rGO can act as the electron donors or as reductants to produce metal nanoparticles from precursors [14]. However, the reductive capability of N-rGO alone is rather limited and cannot completely convert Au precursors into nanoparticles within a short reaction time (*τ* = 1.0 s) at ambient temperature. The presence of excess NaBH_4_ could compensate this reduction power, leading to the generation of nano-Au catalysts immediately after coming into contact with the previously anchored Au salts.

**Figure 2:**
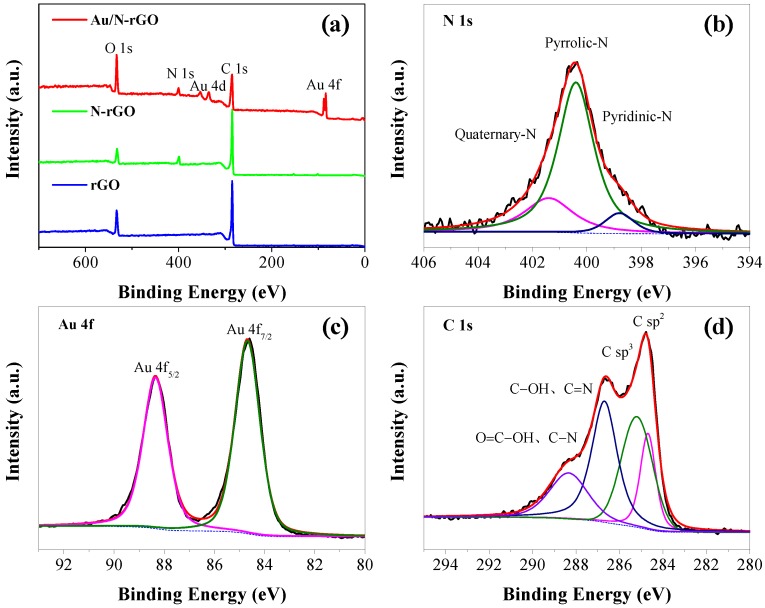
(**a**) X-ray photoelectron spectroscopy (XPS) spectra of as-synthesized Au/N-rGO, N-rGO, and rGO filters. (**b**–**d**) Correspond, respectively, to the XPS spectra of N, Au, and C elements in the Au/N-rGO filter at an Au/C ratio of 0.2%.

XPS was further used to confirm the change in elemental compositions and the oxidation state after N-doping and Au loading. As displayed in Figure 2a, characteristic peaks of C 1s, O 1s, and N 1s were identified from the survey pattern of an N-rGO filter sample, whereas only C 1s and O 1s were observed for the rGO filter. These data provided supportive evidence for the successful doping of N. A decrease in the oxygen content and an increase in the carbon content were observed after Au loading onto the N-rGO. This can be due to the reduction of the oxylated functional groups by NaBH_4_. As determined from the XPS data, the atomic ratio of N in the N-rGO filter was 7.8%, within the typical doping range of 1–15% [37,38,39,40]. From the high-resolution N 1s scan (see Figure 2b), the signal could be further deconvoluted into pyridine nitrogen, pyrrolic nitrogen, and quaternary nitrogen. Based on the result of the peak area integration, the pyrrolic N centered at 399.6 eV (70.6%) was found to be the dominant N in the Au/N-rGO filter, which was higher than that of quaternary N at 401.5 eV (22.5%) and pyridinic N at 398.4 eV (6.9%). The ratio of pyrrolic N:pyridinic N:quaternary N is determined to be 1:0.32:0.10 based on the XPS data. This indicated that the dominant N state was pyrrolic N in the Au/N-rGO filter. Introducing N atoms into rGO could produce more defects and active sites onto which the Au precursors could be easily attached. The atomic ratio of Au was determined to be 0.13%, in agreement with the feeding amount of Au precursors (Au/C = 0.2%). Specifically, the high-resolution scan of Au 4f indicates Au 4f_7/2_ and Au 4f_5/2_, respectively, centered at a binding energy of 84.7 eV and 88.4 eV (peak-to-peak distance was 3.7 eV). This suggested the conversion of Au(III) to Au (0) by NaBH_4_ reduction. The asymmetric C1s spectrum of N-rGO (see Figure 2d) could be further deconvoluted into four characteristic peaks. Among them, the peaks centered at 284.7, 285.3, 286.5, and 288.3 eV corresponded to the C sp^2^, C sp^3^, C–OH, and C=N bonds, as well as O=C–OH and C–N bonds, respectively.

### Catalytic Performance Evaluation

**Figure 3:**
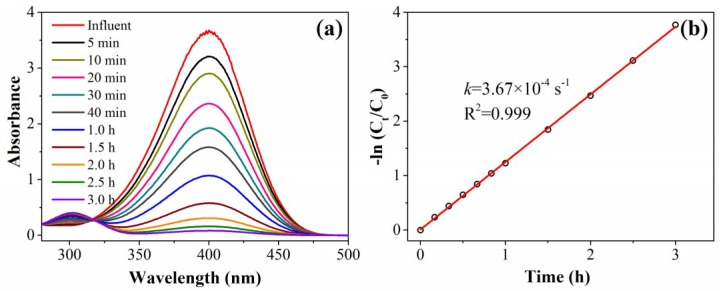
(**a**) Time-dependent UV-Vis absorption spectra and (**b**) rate kinetics of the Au/N-rGO filter (Au/C ratio of 0.2%) catalyzing 4-nitrophenol (4-NP) reduction into 4-aminophenol (4-AP) in a batch reactor system.

To evaluate the performance of the as-designed catalytic filter, the authors used the conversion of 4-nitrophenol (4-NP) into 4-aminophenol (4-AP; using NaBH_4_ as the reductant) as a model catalytic reaction. As a control experiment, the time-dependent UV-vis absorption spectra of 4-NP in the presence of a typical Au/N-rGO filter (prepared by using 50 mL 0.04 mmol/L HAuCl_4_ solution and 50 mL 0.5 mmol/L NaBH_4_ solution passed through the 5 mg of N-rGO filter sequentially) and excess NaBH_4_ (250 excess times) in a conventional batch reactor are shown in Figure 3. The characteristic 4-NP absorption peak, centered at 400 nm, gradually decreased with time due to the continuous catalytic conversion to 4-AP in the presence of an Au/N-rGO catalyst. Consequently, the characteristic 4-AP absorption peak, centered at 300 nm, emerged and increased with time. The yellow 4-NP solution was completely converted to colorless 4-AP after 3 h continuous reaction. A linear relationship between ln(*C*/*C*_0_) and reaction time was obtained. These data led to pseudo-first-order kinetics with a rate constant, *k_app_*, calculated to be 3.67 × 10^−4^ s^−1^. In this study, the nano-Au catalysts were embedded onto/into the N-rGO filter surface. The reactants needed more time to diffuse and get in contact with these active sites, whereas the interaction of AuNPs and 4-NP molecules was much easier by Brownian motion in a homogeneous system. This could explain why the *k_app_* obtained in this study was only 24% of that obtained employing the AuNPs catalyst in a homogeneous batch system [20].

**Figure 4:**
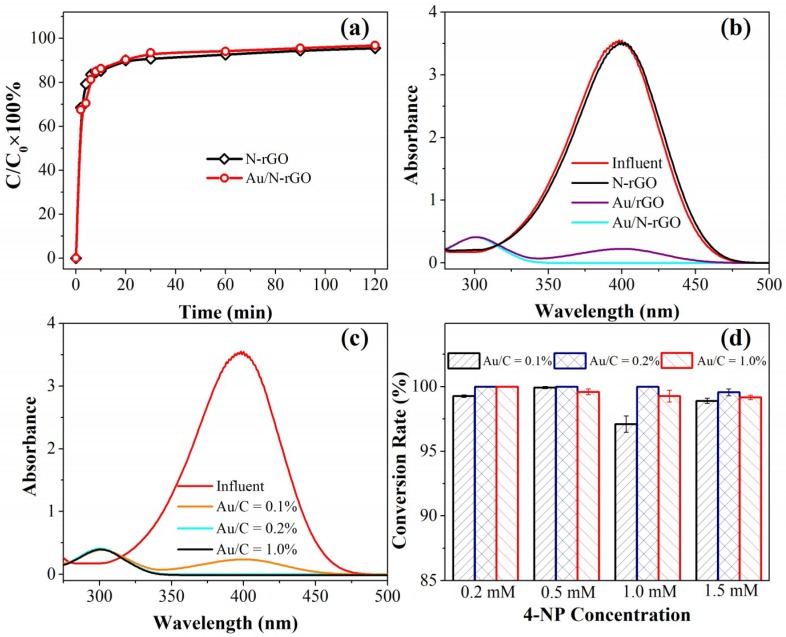
(**a**) Breakthrough curves of the N-rGO filter and the Au/N-rGO filter; (**b**) UV-Vis absorption spectra before and after passing through the N-rGO filter, the Au/rGO filter, and the Au/N-rGO filter; (**c**) effect of Au loading amount on 4-NP conversion by the Au/N-rGO filters, where the Au/C ratio is 0.1%, 0.2%, and 1.0%; (**d**) the 4-NP conversion as a function of Au loading and 4-NP concentration.

For comparison, the authors performed the conversion of 4-NP in a continuous flow catalytic filter system. A continuous flow catalytic filter design is more advantageous and attractive compared with a conventional batch reactor. To eliminate adsorption on the removal of 4-NP, the adsorption saturation of 4-NP was first performed by passing 0.2 mmol/L 4-NP alone through the filter at a flow rate of 1.0 mL/min. As shown in Figure 4a, similar breakthrough curves were obtained for N-rGO and Au/N-rGO filters. This indicated that nano-Au loading posed a negligible effect on the adsorption behavior of the N-rGO filters as well as on the mass transfer-controlled sorption process. Because of the limited thickness (4.0 ± 0.3 µm, as shown in Figure S3) of the N-rGO filter and the convection-enhanced mass transport, the effluent 4-NP concentration increased rapidly in the first 10 min and a complete breakthrough occurred over 40 min continuous filtration for both filters. Another reason for this rapid 4-NP sorption may be due to the strong π–π interactions between the two-dimensional planar hexagonal carbon structure of rGO and the aromatic molecular structure of 4-NP.

After the 4-NP sorption saturation, a certain amount of 4-NP passed through the catalytic filter together with NaBH_4_. Some control experiments were performed to clarify the contribution of nano-Au and/or doped N on the 4-NP conversion. As shown in Figure 4b, one control experiment using the N-rGO filter alone exhibited negligible 4-NP conversion under similar conditions. N-rGO has been reported to possess good catalytic activity toward selected oxidation and reduction reactions [41,42,43]. However, the N-rGO only demonstrated limited activity toward the 4-NP conversion reaction, and this confirmed the essential role of the nano-Au catalyst. Another control experiment using the Au/rGO filter with an Au/C ratio of 0.2% highlighted the important role of doped N. Under similar experimental conditions, an Au/rGO filter could only partially convert 0.2 mmol/L of 4-NP, whereas an Au/N-rGO filter could completely convert 4-NP to 4-AP by a single pass (*τ* = 1.0 s) through the catalytic filter. After N doping, more Au precursors carrying opposite charges could be adsorbed, and a uniform Au distribution could be expected on the N-rGO surface due to the uniform doping of nitrogen. This could provide more active sites on the catalytic filter. Compared to a batch reactor system, the continuous flow catalysis significantly promoted the catalytic performance even when employing the same amount of Au catalyst. The evidently enhanced catalytic kinetics in the catalytic filter could be ascribed to the convectively-enhanced mass transport during filtration. The high-performance nanoscale catalysts were strongly anchored onto the hosting support, ensuring a long-term stability and catalytic activity. In a conventional batch system, the activity of the nano-Au catalysts were predominantly controlled by diffusion, which greatly limits the mass transport performance and leads to sluggish catalytic kinetics. Alternatively, the catalytic reaction and separation of the catalyst from the solution (including the product) take place simultaneously by employing a catalytic filter design. Thus, the post-separation of catalysts can be avoided and the mass transport in a continuous-flow design can be enhanced convectively using an external peristatic pump.

Some key operational parameters were further optimized. First, the nano-Au loading amount was an important parameter in the current catalytic filter system. As shown in Figure 4c, under the experimental conditions of 1.0 mL/min flow rate, 0.2 mmol/L 4-NP, and 50 mmol/L NaBH_4_, the complete 4-NP conversion was obtained once the Au/C ratio was higher than 0.2%. Only a partial conversion of 4-NP was obtained at a lower Au/C ratio of 0.1%, indicating that sufficient active sites were available for the catalytic conversion of 4-NP. These results were expected, as the Au nanoparticles in the filter provided active sites for the reaction. In general, a higher Au loading amount could produce more catalysts for the system. Furthermore, increasing the 4-NP concentration could also lead to an incomplete 4-NP conversion. For example, at an Au/C ratio of 2.0%, only an incomplete conversion was observed at a 4-NP concentration of 1.5 mmol/L. However, at an Au/C ratio of 5.0%, a complete 4-NP conversion was only be obtained at 0.2 mmol/L of 4-NP. The effect of the 4-NP concentration could be explained by the mass transport limitation in the current catalytic filter system. A higher substrate concentration may lead to insufficient contact between 4-NP and the active sites due to a limited contact time between active sites (i.e., nano-Au catalyst) and substrate (i.e., 4-NP). Furthermore, a proper Au loading was necessary since the catalytic performance at an Au/C ratio of 0.2% was found to be higher than that of 0.5%. This phenomenon could be explained by the size effect of the nano-Au catalyst. As suggested by the FESEM and TEM characterizations of the Au/N-rGO filters with different Au loadings, the nano-Au size increased with the Au precursor concentration, and agglomeration occurred at the higher Au loading (e.g., Au/C ratio of 0.5%). Generally, the catalytic activity of nano-Au was opposite to their particle size. Although the particle size at 0.1% Au/C ratio is the smallest, the limited amount of nano-Au produces insufficient catalytic activity of the catalytic filter. To further verify the stability of the as-prepared catalytic filters, 5 h of continuous filtration of 1.0 mM 4-NP demonstrated a negligible decline in conversion efficiency, indicating an excellent catalytic activity and stability of the catalytic filters (as shown in Figure S5).

**Figure 5:**
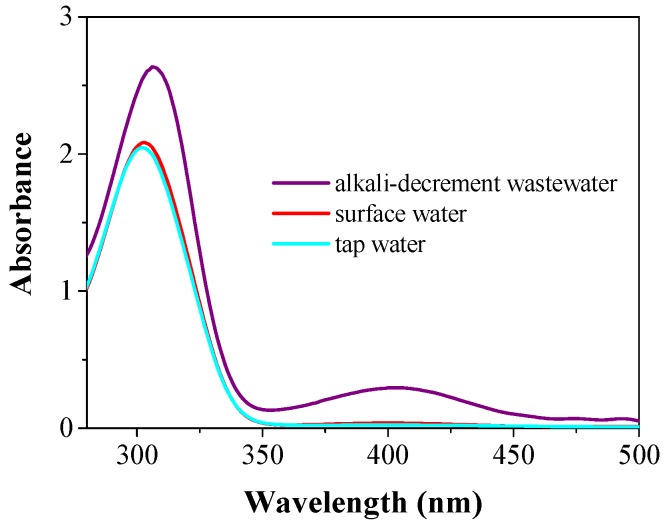
UV-Vis absorption spectra of three 4-NP spiked real water samples after a single passing through an Au/N-rGO filter. Experimental conditions: 0.2% Au/C ratio, 1.0 mL/min flow rate, 1.0 mM 4-NP, and 250-fold excess NaBH_4_.

To further evaluate the performance of the catalytic membrane system with respect to real water samples, 1.0 mmol/L 4-NP and 250-fold NaBH_4_ (compared with 4-NP) were spiked into real water samples collected from a local tap, a local river, and industrial dyeing wastewater (alkali-decrement wastewater, diluted 1000 times), respectively, before filtration. The influent spectra of the three 4-NP spiked real wastewater samples are available in the Supplementary Materials (see Figure S6). As shown in Figure 5, the catalytic kinetics in all three real water samples displayed a similar behavior to that in DI-H_2_O, and a complete 4-NP conversion was achieved in tap water and reservoir water. A >98.3% 4-NP conversion was also achieved under the dyeing wastewater conditions (COD_Cr_: 37,668.4 mg/L). It is noteworthy that the three real water samples had totally different compositions and characteristics. These results suggested an excellent catalytic activity in the as-developed catalytic membrane system. In addition, hydrogen may be produced from the reaction of NaBH_4_ and H_2_O and a large quantity of hydrogen gas may block the filter pores and significantly increase the internal pressure within the filtration device. To slow down the kinetics for hydrogen gas production, the 4-NP solution was stored in ice water before the addition of NaBH_4_. The catalytic activity of the filter may also be affected once it is allowed to dry out. The shrinkage of the support materials may deteriorate the catalytic performance, and these engineering issues should be resolved before implementing practical engineering applications.

## 4. Conclusions

In conclusion, the authors developed and rationally designed a high-performance catalytic filter for continuous flow catalysis applications. The ligand-free nano-Au catalyst with tunable size and loading was easily loaded onto an rGO “host” by in situ reduction. In the design, the N-rGO served as a superior support with abundant defects. The rational rGO doping with N offered available nucleation sites for the anchoring of Au precursors and the production of nano-Au catalysts. The results demonstrated that this continuous flow catalytic system has evidently enhanced catalytic kinetics compared to a conventional batch system. This design strategy has potential for the fabrication of advanced catalytic membrane systems for use in industrial applications.

## Supplementary Materials

The following are available online at http://www.mdpi.com/2079-4991/8/9/688/s1, Figure S1: FESEM (c–f) images of Au/N-rGO composites with different Au loading amount. Au/C ratio is 0.5% (a), 1.0% (b), 2.0% (c), and 5.0% (d), respectively; Figure S2. FESEM images for cross sectional analysis of Au/N-rGO filter; Figure S3. XRD diffraction patterns of pure rGO, pure N-rGO and Au/N-rGO filters (a); Raman spectra recorded from GO, rGO, N-rGO and Au/N-rGO samples (b); Figure S4. The UV spectra of the three simulated actual wastewater samples after 10 times dilution, Table S1: Elemental analysis of the Au/N-rGO filters with different Au loading amount determined from EDX analysis.
