# Supplementary material for: Ligand-Free Nano-Au Catalysts on Nitrogen-Doped Graphene Filter for Continuous Flow Catalysis
Source: Nanomaterials (Basel). 2018 Sep 5;8(9):688. doi: 10.3390/nano8090688 (PMC6165004; doi:10.3390/nano8090688)
Supplement: Supplementary file 1 [file nanomaterials-08-00688-s001.pdf]

nanomaterials

Ligand-Free Nano-Au Catalysts on Nitrogen-Doped Graphene Filter for Continuous Flow Catalysis

Yanbiao Liu [1,2,*], Xiang Liu [1], Shengnan Yang [1], Fang Li [1,2], Chensi Shen [1,2], Chunyan Ma [1], Manhong Huang [1,2] and Wolfgang Sand [1,3]

1 Textile Pollution Controlling Engineering Center of Ministry of Environmental Protection, College of Environmental Science and Engineering, Donghua University, Shanghai 201620, China
2 Shanghai Institute of Pollution Control and Ecological Security, Shanghai 200092, China
3 Institute of Biosciences, Freiberg University of Mining and Technology, Freiberg 09599, Germany
* Correspondence: yanbiaoliu@dhu.edu.cn; Tel.: +86-21-67798752

Supporting Information: 1 table and 6 figures.

Table S1. Elemental analysis of the Au/N-rGO filters with different Au loading amounts determined from Energy-dispersive spectroscopy (EDS) analysis.

Au/N-rGO	Element	Weight %	Atomic %
Au/C = 1.0%	C K	62.8	77.2
	Au K	7.0	0.5
Au/C = 2.0%	C K	58.5	72.0
	Au K	13.7	1.3
Au/C = 5.0%	C K	50.6	78.8
	Au K	34.9	3.3

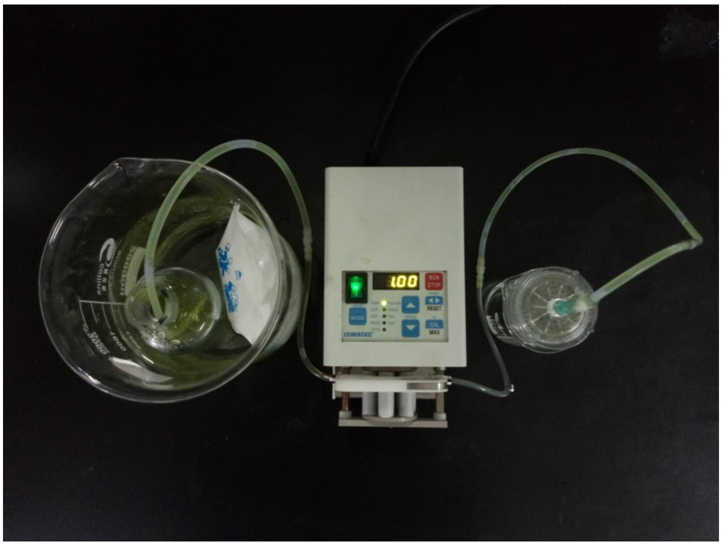

Figure S1. The photograph of operational device in flow catalytic system.

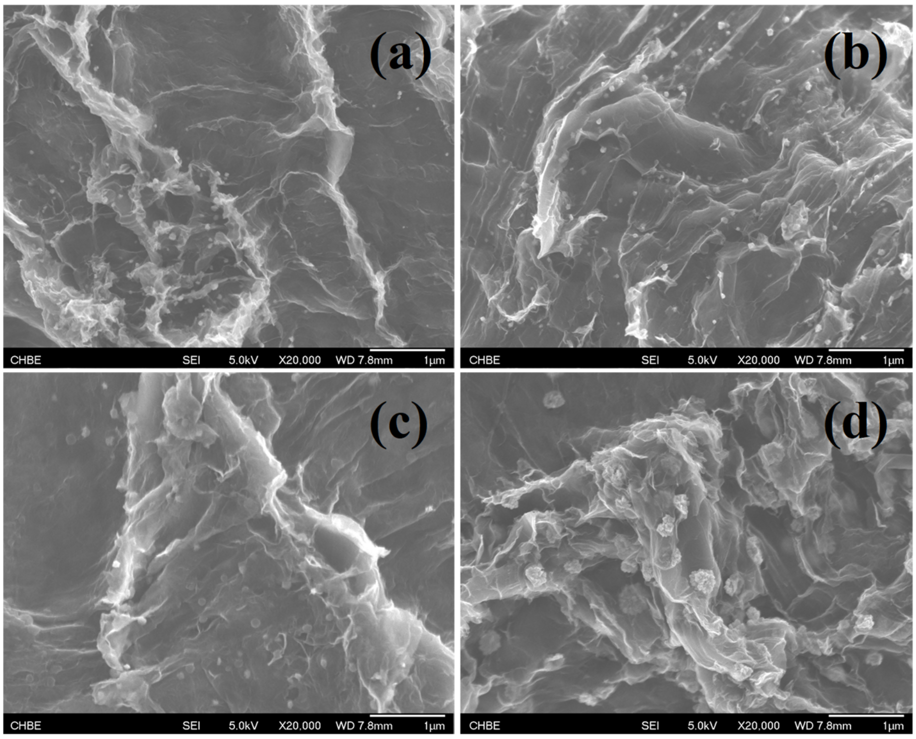

Figure S2. Field emission scanning electron microscopy (FESEM) images of gold/nitrogen-doped graphene (Au/N-rGO) composites with different Au loading amounts. Au/C ratio is (**a**) 0.5%, (**b**) 1.0%, (**c**) 2.0%, and (**d**) 5.0%, respectively.

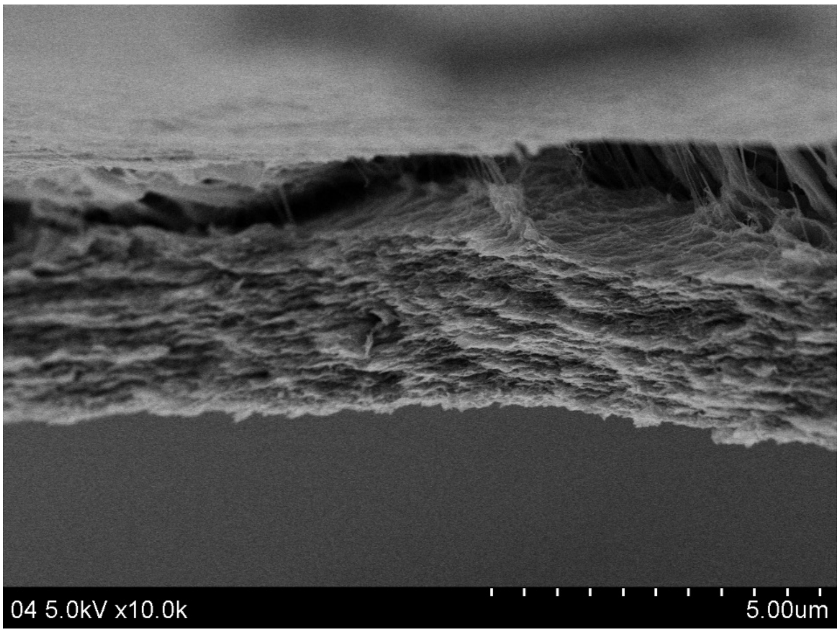

Figure S3. FESEM images for cross sectional analysis of Au/N-rGO filter.

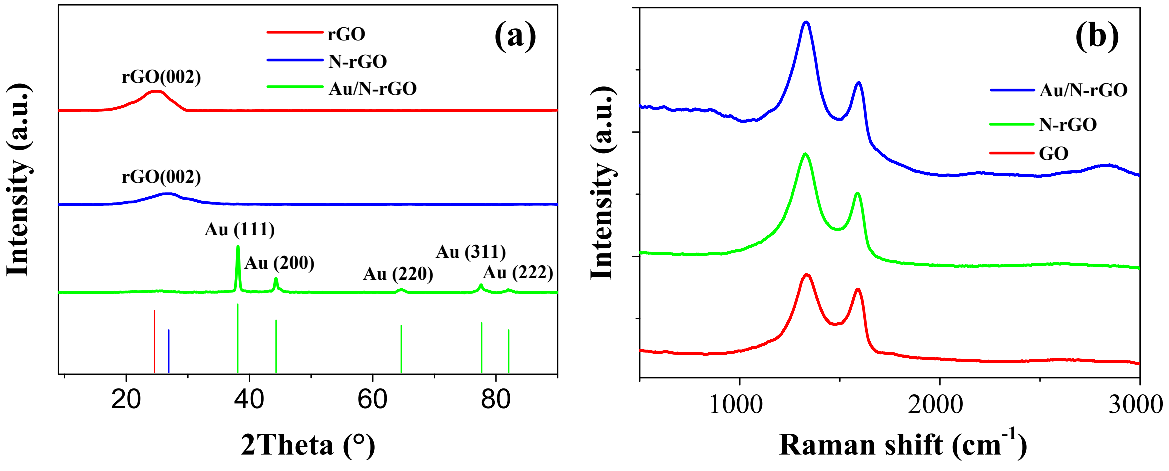

Figure S4. XRD diffraction patterns of pure rGO, pure N-rGO and Au/N-rGO filters (**a**); Raman spectra recorded from GO, N-rGO and Au/N-rGO (Au/C=0.2%) samples (**b**).

The typical XRD patterns of the as-prepared pure rGO, pure N-rGO, and Au/N-rGO (Au/C = 0.2%) filters are shown in Figure S4a. The obtained XRD patterns of the Au/N-rGO filter unequivocally confirmed the presence of Au particles in the reduced graphene. Specifically, in the case of the Au/N-rGO sample, the five reflections at 2θ angle of 38.1°, 44.3°, 64.6°, 77.6°, and 82.0° were observed and could be assigned to the (1 1 1), (2 0 0), (220), (3 1 1), and (2 2 2) crystal planes with a face-centered cubic structure of AuNPs, respectively. Furthermore, the broad XRD intense characteristic peaks centered at 2θ angle of 23.0° and 24.7°, originating from the movement of the (002) peak of the initial graphene oxide due to the elimination of intercalated water molecules between neighboring rGO sheets, could both be assigned to the (002) plane of the reduced graphene oxide. This broad XRD signal of rGO indicated the poor ordering of sheets along the structure direction. It also demonstrated that the structure of the rGO was composed of the few-layer stacked sheets, which was consistent with the results obtained by SEM imaging. After the anchoring of the AuNPs, the diffraction intensity of the (002) crystal plane of rGO was distinctly decreased, which could be attributed to the deposition of Au nanoparticles on the surface of the reduced graphene oxide. No other characteristic peaks from the rGO and N-rGO could be observed at the range of 2θ in Figure S4a, most probably because of strong interference from the polytetrafluoroethylene (PTFE) support and/or limited thickness of the as-synthesized filters. The Raman spectra recorded from the GO, N-rGO and Au/N-rGO (Au/C = 0.2%) samples are shown in Figure S4b. The Raman spectra exhibited characteristic D peaks and G peaks at ~1350 cm^{-1} and ~1580 cm^{-1}, respectively. The prominent D peaks arose from the structural imperfections created by the attachment of hydroxyl and epoxide groups on the carbon–carbon bonds in a basal plane of GO. The G peaks were an indicator of the stacking structure. It is evident that the intensity in the Raman spectrum of Au/N-rGO was higher than that of only N-rGO due to the surface-enhanced Raman scattering (SERS) activity of the graphene and Au nanoparticles, which also provided evidence for the successful loading of Au. Meanwhile, the Raman spectra of N-rGO and Au/N-rGO are similar, indicating that the loading of AuNPs does not greatly reduce the sizes of in-plane sp^2 domains of graphene and damage the structure of the graphene sheets.

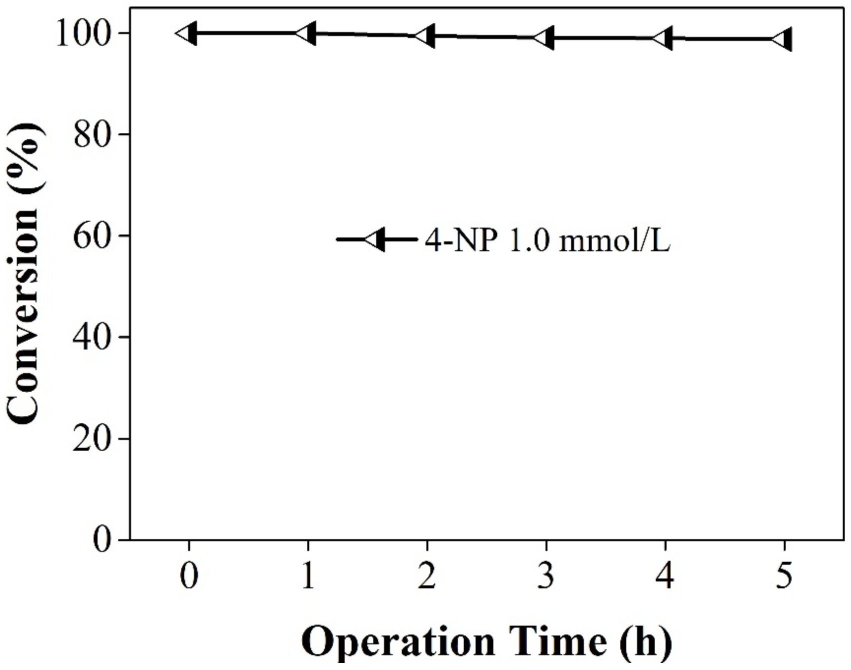

Figure S5. Effect of operation time on the catalytic performance of an Au/N-rGO (Au/C=0.2%) membrane.

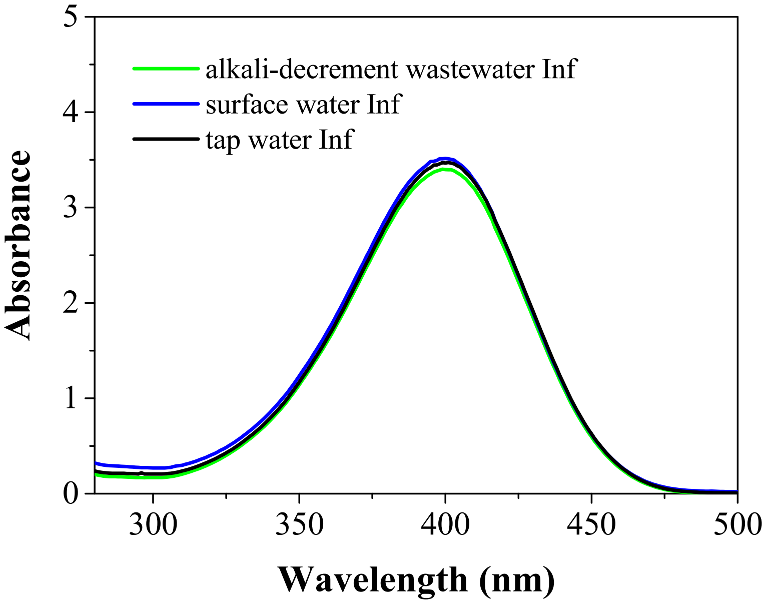

Figure S6. UV spectra of the three simulated real wastewater samples diluted 10 times.

© 2018 by the authors. Submitted for possible open access publication under the terms and conditions of the Creative Commons Attribution (CC BY) license (http://creativecommons.org/licenses/by/4.0/).
